# Non-invasive stroke volume estimation by transthoracic electrical bioimpedance *versus* Doppler echocardiography in healthy volunteers

**DOI:** 10.1080/03091902.2019.1599074

**Published:** 2019-04-15

**Authors:** Mirae Harford, Samuel H. Clark, Jodie F. Smythe, Stephen Gerry, Mauricio Villarroel, Joao Jorge, Sitthichok Chaichulee, Lionel Tarassenko, Duncan Young, Peter Watkinson

**Affiliations:** a Critical Care Research Group, Nuffield Department of Clinical Neurosciences, University of Oxford, Oxford, UK;; b Department of Engineering Science, Institute of Biomedical Engineering, University of Oxford, Oxford, UK;; c Critical Care Department, University College London Hospitals NHS Foundation Trust, London, UK;; d Adult Intensive Care Unit, John Radcliffe Hospital, Oxford University Hospitals NHS Foundation Trust, Oxford, UK;; e Centre for Statistics in Medicine, University of Oxford, Oxford, UK

**Keywords:** Stroke volume, echocardiography, thoracic bioimpedance

## Abstract

Thoracic electrical bioimpedance (TEB) and transthoracic echocardiography (TTE) are non-invasive methods to estimate stroke volume (SV) and cardiac output (CO). Thoracic electrical bioimpedance is not in widespread clinical use with reports of inaccurate cardiac output estimation compared to invasive monitors, particularly in non-healthy populations. We explore its use as a trend monitor by comparing it against thoracic echocardiography in fifteen healthy volunteers undergoing two physical challenges designed to vary cardiac output. Of all paired values, 54.6% showed gross trend agreement and only 1.9% showed direct disagreement between the two monitors. Our results show thoracic bioimpedance may have a role as a non-invasive cardiac output trend monitor in healthy volunteer studies.

## 1. Introduction

Thoracic electrical bioimpedance (TEB) cardiac output (CO) measurement has been validated against thermodilution measurements in healthy volunteers [1] but has been reported to be unreliable in clinical settings and is not in widespread clinical use [2]. In contrast, the use of transthoracic echocardiography (TTE) for cardiac functional assessment is increasing, particularly in critical care [3].

We designed a healthy volunteer study primarily to compare video-derived proxies for CO with TEB and TTE. These reference methods were chosen for their non-invasive nature and their validated performance in healthy subjects against invasive monitors. TEB can give continuous estimates of stroke volume (SV), whereas TTE can provide intermittent image-based estimate.

In this report, we describe the agreement in SV measurements between TEB and TTE. We compared raw SV measurements (rather than the CO – the product of SV and heart rate derived from these measurements) in order to eliminate potential compensatory mechanisms and assess the raw values. The reliability of TEB as a monitor is important to explore given its ability to estimate SV in a quick, non-invasive manner and its inter-observer reproducibility [4]. If TEB showed good agreement with TTE, its ability to provide continuous SV assessment following TTE assessment and calibration would be useful in healthy volunteer trial settings. It may also provide justification for further studies exploring TEB as a monitor of SV changes in other populations.

## 2. Materials and methods

This study was carried out in accordance with the Declaration of Helsinki and approved by the Oxford University Research and Ethics Committee/Clinical Trials and Research Governance (R45629/RE003) and Oxford University Hospitals NHS Foundation Trust ethics board (R&D reference:12056). Written informed consent was obtained from all participants. This manuscript adheres to the applicable EQUATOR guidelines. ISRCTN registration: ISRCTN76998049 .

We recruited male subjects to facilitate simultaneous image recording from multiple exposed regions of skin. A CardioScreen 1000 device (Medis, Ilmenau, Germany) was used to measure SV by TEB. The device was attached to the subject following the manufacturer’s instructions, including an arterial compliance modulation (ACM) sensor to measure arterial pulse waves *via* earlobe photoplethysmography.

A qualified cardiac sonographer acquired TTE video clips at one-minute intervals using Philips CX50-US system (Philips Ultrasound Systems, Bothell, WA). TTE was not acquired during exercise as good quality images are not possible during movement. Two blinded cardiac sonographers made SV measurements from the images offline. The mean of the two values was used for each image. Inter-rater reliability was estimated by calculation of inter-observer variability [5]. TTE values with no corresponding TEB value (e.g., due to signal loss) were excluded from paired analysis.

The experimental period began with a 10-min rest period during which baseline data were collected. Each subject performed exercise for 10 min with set resistance (100 watts at 60 rpm) using a pedal exerciser. After a 10-min rest, subjects immersed their hand in ice water for 5 min or until the point of pain, following the original description of the cold pressor test (CPT) [6]. We continued monitoring for a further rest period of 10 min. We defined periods of interest as the 5 min immediately following cessation of exercise, and the full duration of CPT.

The manufacturer’s software (Cardio Vascular Lab, Medis, Ilmenau, Germany – using the Bernstein and Sramek [7] method for SV estimation) was used to record and export TEB data. We exported data to a spreadsheet and used a statistical package for analysis (Microsoft Excel 2010, Redmond, WA and IBM SPSS Statistics for Windows version 22.0, IBM Corp. Armonk, NY).

### 2.1. Numerical and statistical methods

For TTE the SV was calculated from the aortic root velocity time integral and aortic root diameter. We averaged three heartbeats, noting the time of the first heartbeat measured. For TEB we calculated average SV from all the heartbeats in a 10-s window centred on this time. We compared the raw TTE and TEB SV estimations using Bland–Altman methods, correcting for multiple measurements from each subject [8].

We compared the direction and amplitude of changes in SV from baseline measured by each monitoring device in order to gauge the utility of TEB and TTE as trend monitors. The resting SV for each subject was calculated for each device as the arithmetic mean of SV during the initial resting phase, the last 5 min of post-exercise rest and the last 5 min of post-CPT rest. All measurements were then transformed to the deviation from the calculated resting SV for the device. We created a sub-group of the dataset from the periods of interest. We analysed trend agreement in two ways.

We standardised the values reflecting changes from baseline as a percentage using Equation (1).

(1) $$\text{Standardised deviation resting SV}=\;\frac{\text{Measured value}\;-\text{ Resting SV}}{\text{Resting SV}}\times 100.$$

We used the method of Montenji et al. [9] to analyse the standardised deviation values. This “clinical concordance method” uses four types of trend agreement when comparing CO monitor devices. Good trend agreement is when two measurements change in the same direction to a similar or varying extent. Poor trend agreement is when one measurement does not change or the directions of change are opposite. We also performed linear regression analysis comparing transformed TEB and TTE values. Both methods were undertaken for all paired analyses and for only the periods of interest.

## 3. Results

Fifteen healthy volunteers aged 20–38 years were recruited and included in the final analysis. Raw data plots are shown in Supplemental Figure 1.

The mean inter-observer variability for SV measurement using TTE was 10 ml (SD 10 ml). Out of 708 echocardiography image measurements, 3 had inter-observer measurement variability greater than 30% and were excluded from analysis. Twenty-four (3.4%) of the remaining 705 images had no corresponding TEB value due to signal loss and were excluded from analysis (681 paired measurements with TEB). Of these paired measurements, 155 lay within defined periods of interest.

The Bland–Altman plot of the agreement between the two SV measurement methods showed a mean bias of +31 ± 37 ml (mean ± limits of agreement) towards TEB (Supplemental Figure 2). The Bland–Altman plot comparing raw SV measurements was funnel-shaped, implying a better agreement around the physiological range of 60–90 ml and reduced agreement as SV increased above 90 ml.

**Figure 1.**
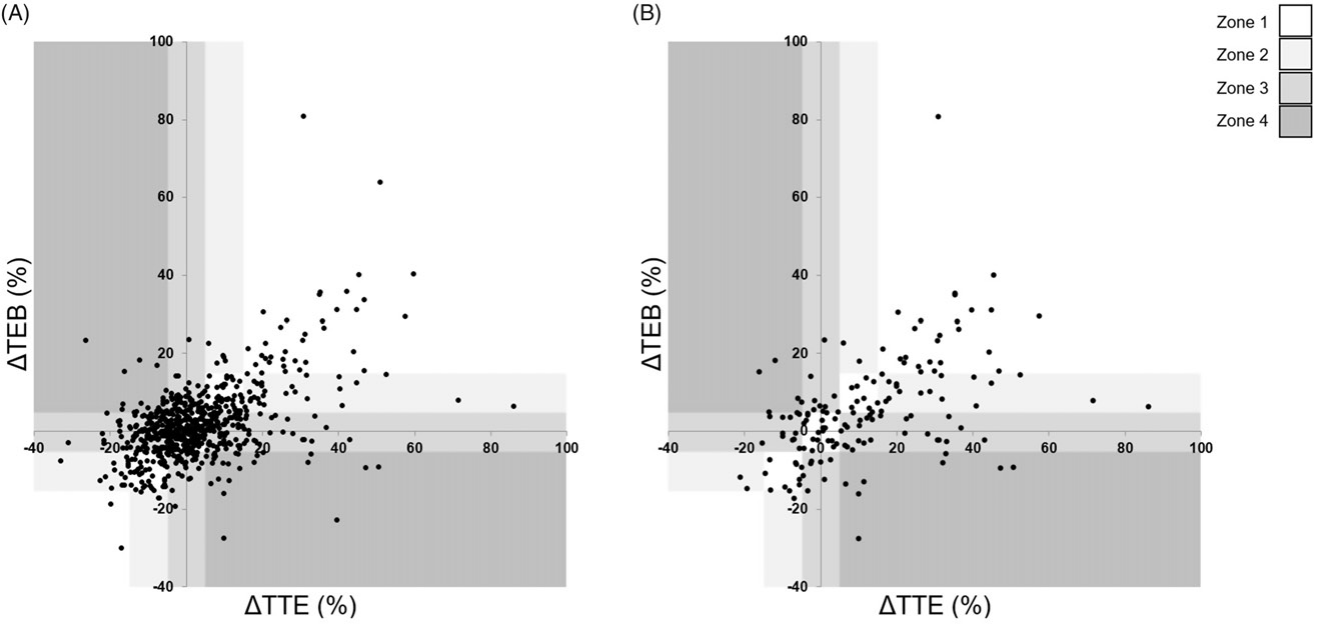
Echocardiographically (TTE) determined *versus* bioimpedance (TEB) determined percentage stroke volume deviation from resting stroke volume for all paired measurements (1(A)) and period of interest only (1(B)) with clinical concordance and error grid plots. Zone 1 indicates the clinical concordance categories in which SV measured by the two methods change in the same direction and to the same extent. In zone 2, they change in the same direction but not to the same extent. In zone 3, one measurement changes (>5%) while the other is constant (<5%). Zone 4 represents opposite changes using the two methods [9].

Figure 1(A) shows the comparison of percentage deviation from resting SV for all measurements. Figure 1(B) shows the same comparison for the 155 values within the periods of interest (with resting values removed). The concentration of data points surrounding the origin in Figure 1(A) are fluctuations around the mean resting SV during the resting phases, indicated by their absence in Figure 1(B). The distribution of measurements assessed using the clinical concordance method [9] is indicated by the shaded zones in Figure 1. Of all paired values 54.6% fell within zones 1 or 2 indicating gross trend agreement. This agreement was stronger during periods of interest (63.2%). Only 1.9% of the full dataset (3.9% of measurements during periods of interest) fell within zone 4 which indicates direct disagreement between the two monitors.

The gradient of the regression line for all measurements (Figure 1(A)) was 0.41 (95% CI 0.36 to 0.45) with an intercept of 0.31% (95% CI −0.39 to 1.02), *r* = 0.54. The gradient of the regression line for periods of interest (Figure 1(B)) was 0.45 (95% CI 0.34 to 0.57) with an intercept of 0.2% (95% CI −2.6 to 2.9), *r* = 0.53.

A Bland–Altman plot of the standardised values shows bias of +2% (towards TTE) with limits of agreement −23 to 27%. Linear regression shows a positive slope with a gradient of 0.36 (95% CI 0.28 to 0.44, *r* = 0.32, *p* < .001) (Supplemental Figures 4 and 5).

Linear regression analysis of the deviation from resting SV pre-standardisation for all measurements show a correlation of *r* = 0.54 (*p* < .001). The gradient of the regression line was 0.41 (95% CI 0.36 to 0.45) with an intercept of 0.31 ml (95% CI −0.39 to 1.02). Linear regression analysis for measurements during periods of interest showed a correlation of *r* = 0.53 (*p* < .001). The gradient of the regression line was 0.66 (95% CI 0.49 to 0.84) with an intercept of 0.5 ml (95% CI −2.04 to 3.04) (Supplemental Figure 3).

## 4. Discussion

Our study results show a clear trend agreement between SV changes detected by TTE and TEB in healthy subjects. Our findings differ from those of a previous study by Fellahi et al. [10] which reported unreliable TEB performance in detection of CO changes during positive end-expiratory pressure (PEEP) application and lower body positive pressure. Since the study by Fellahi et al. [10] the manufacturers have incorporated the ACM to the TEB monitor. This measurement of the arterial waveform with additional estimation of the timing of aortic valve closure may have improved SV estimation. Furthermore, the interventions to change SV used in the previous study may have confounded the results. The application of PEEP and lower body positive pressure both affect CO by increasing venous return. Increasing the venous return would be expected to increase the intrathoracic venous volume. This may have overshadowed any simultaneous changes in SV and aortic volume, affecting CO estimation using the bioimpedance method. In contrast, our methods of inducing SV change by simulating sympathetic response (exercise) and peripheral vasoconstriction (CPT) would not be expected to cause large changes in venous return.

Our study has a number of limitations. We carried out the study in a narrow spectrum of healthy volunteers, limiting the application of our results to the same population. Our demonstration of agreement in a narrow spectrum of patients is an important first step, showing agreement can be found. As a result, a wider study including older subjects who may have different intrathoracic vessel compliance and female subjects with different thoracic wall composition (which may affect thoracic impedance) is now needed. Acquiring TTE images and measurements during the exercise period was technically challenging and paired data during this period of increasing SV was not achievable. Instead, we showed agreement in the immediate post-exercise recovery period. Finally, some changes may have occurred between TTE measurements at 1-min intervals. More frequent or continuous echocardiographic estimation may have been valuable. This is particularly true during CPT where the directions of the changes varied across subjects and sometimes included both an increase and a decrease within the 5-min periods.

Our results show bioimpedance follows changes in CO in healthy volunteers, with only around 4% of measures being in the opposite direction to those found using echocardiography. Bioimpedance was able to detect when a significant change had occurred and in what direction, although it was less good at quantifying the degree of change. This is an improvement on previous findings, suggesting that TEB may be developed to have a role as a non-invasive CO trend monitor in healthy populations. The results of this study cannot be extrapolated to non-healthy populations but provides justification for further TEB studies in wider population groups.

## Data Availability

The data that support the findings of this study are available from the corresponding author, MH, upon reasonable request.
